# The Effect and Result of Sero-therapy in Tuberculosis

**Published:** 1896-07

**Authors:** Paul Paquin

**Affiliations:** St. Louis, Mo.


Contributed to the Texas Medical Journal.

### THE EFFECT AND RESULT OF SERO-THERAPY IN TUBERCULOSIS.

BY PAUL PAQUIN, M. D., ST. LOUIS, MO.

[Written for the Texas State Medical Association, April, 1896.]

THAT SERO-THERAPY in tuberculosis of any form is entirely within scientific bounds, and based on scientific and practical grounds, is clearly demonstrated by the results obtained abroad and in this country, both by experiments and clinical tests. As I have before expressed, the serum treatment of tuberculosis may be said to date back as far as 1890. Since that year, Bernheim, Bertin, Picq, Coupan, St. Hiliare and Bahes experimented with goat blood and dog blood, and a little with the serum of these animals in the treatment of pulmonary tuberculosis. Maragliano experimented with the serum of the blood of the ass in the treatment of consumption. Richet and Hericourt also experimented with some form of anti-tubercle serum, but with scant success. It was reserved for this country to *immunize* the *horse* against tuberculosis, and produce a successful anti-tubercle serum. I began my experiments on this line long ago, and applied sero-therapy in man in 1894, publishing my first results early in 1895. The immunization consists in rendering the animal resistant to the reaction produced by the bacilli and their toxine in his system. Detailed accounts of the process have been published already. Before this immunization in 1894 and 1895, and the distribution of serum among the doctors throughout this country, there had never been any general attempt to apply sero-therapy in this disease. Unfortunately for the work of our laboratory and our efforts in establishing the value of anti-tubercle serum in the eyes of a few, the innovation had the disadvantage of being of American birth. It is the sequel to some years of laboratory and field investigations in bacteriology, during my term as bacteriologist and pathologist of the medical department of the State University of Missouri. Our results provoked some encouraging comments, considerable unkind criticism, and even denunciations, chiefly from those who know nothing of the matter, or who had given serum cursory trials; usually in hopeless cases. Many critics seem to have considered it impossible for a general practitioner to accomplish anything valuable in experimental medicine, or to advance abreast of the Europeans in the treatment of pulmonary tuberculosis, by a method depending on careful experimental and clinical labors in the laboratory and in the hospital. Now we have to our credit, *absolute recoveries*, and a large number of improvements, and no less than seventy-five physicians have contributed encouraging reports, ranging from the arrest of the active tubercular process in apparently hopeless cases, to apparently radical cures of early cases.

The therapeutic influence of serum seems to be exercised on those cells of the body whose duty it is to act as scavengers. The serum, in a physiologic way, antagonizes the development of the germs, and neutralizes their toxic products in the system. Thus new means of protection are created, or "the natural means of defense," as Bouchard puts it, "are exalted," and the invading microbes are arrested in their devastation. In other words, just as nature does, in an organism invaded by infection, produce in that organism a defensive power to fight the progress of the invading germ, by which power spontaneous recoveries sometimes occur, so does an animal under process of immunization against infectious diseases, produce in the blood the necessary defensive elements. By laboratory processes these are filtered out, preserved and then used in man; thus saving his system from the dangerous and usually fatal necessity of producing them for his own salvation.

It is evident, therefore, that the anti-toxine, or the therapeutic serum of any kind, produced by nature in man or animal, by the action of a *special germ*, or *its products*, can be expected to neutralize and overcome only this germ and this product. Consequently, in any specific disease with complications of a microbic order, *the complicating* germs are not directly affected by the specific anti-toxine for the primary and specific malady. The complications subside only because of the strength gained in a general way by the organism when the specific germs in question are arrested in their progress. On the other hand, in cases in which the lesions have lowered the vitality of the nervous system, and of the circulation, to a point where the physiologic functions are very seriously impaired, anti-toxines, including anti-tubercle serum, can not accomplish as good results as in infections without great encroachment. It is, therefore, in the earliest stage of any infectious malady that serum must be used to obtain the best results, although it should be used in any stage, it being the only plank of salvation even in most advanced disorganizations. An early diagnosis is consequently an obvious necessity in consumption, which is so often complicated, and must result in irreparable lesions in the great majority of cases.

If we study the results of sero-therapy in diphtheria, in which malady it has given the most striking recoveries, you will notice that it is only in the earlier stage that the most radical and rapid cures have been produced, and that in complicated cases good results have been comparatively meagre. The percentage of recovery in the early stage of diphtheria is no greater than that of the early stage of tuberculosis, only, necessarily, more rapid. The Ann Urban Hospital, one of the largest of the hospitals belonging to the city of Berlin, treated all diphtheria patients in the year 1894-95, twenty of whom were still in the hospital at the end of that year. Two hundred and fifty-five were discharged cured. Of the 245 who were treated with serum, 28 per cent. died, while of the 146 who were treated otherwise, 41.9 succumbed. Of the serum cases, 53.2 per cent. were serious, 23.7 severe, and the rest slight. No injurious sequelæ of the serum were observed, and its salutary effect was *proportionate* to the *earliness* of its application and the strength of the first doses. The general deduction is that the serum is *not an infallible*, but a *highly valuable*, remedy in such diseases. So is anti-tubercle serum. As a matter of fact, in the reports of treatment of pulmonary tuberculosis by the use of serum, only fourteen cases, so far as I know, were in the first stage (none in the incipient stage), and every one of those have recovered; all the others were in an advanced condition of the disease, and the lesions were such as to preclude the proper execution of the physiological functions necessary for the proper action of the serum, except to a limited degree. And still, with all these drawbacks, our serum has to its credit diseases arrested, if not cases of true recoveries, in which there was mixed infection and even cavities, as is shown by the reports.

All that we ask of the physicians in the use of the serum is, that a most careful diagnosis be made, establishing the nature and stage of the disease beyond doubt, thus putting each case on its merits, and treating each with a good knowledge of the existing conditions. It must be understood that serum is not a magic cure; that it will not perform miracles. It is a rational remedy for consumption, and benefits can be expected chiefly in pure cases of tuberculosis before any breaking down has taken place. The results are best at the hands of good, careful doctors, who are persistent, and watch their cases steadily and conscientiously. The following record of cases is self-explanatory, and a good indication of the kind of cases that can be treated with serum successfully:

### ACUTE TUBERCULOSIS.

Miss V. Z. (East St. Louis, Ill.); physician in charge, Dr. J. L. Wiggins, East St. Louis, Ill.; consultants, Drs. J. R. Lemen, of St. Louis, and myself.

Miss V. Z. had been ill several months, and the last weeks previous to consultation she had been prostrated in bed, with a complete history of acute pulmonary tuberculous pneumonia. The range of abnormal temperature was between 103° and 104° continually, for many weeks prior to treatment. Microscopical analysis demonstrated bacilli of tuberculosis. Each physician consulted diagnosed acute tuberculosis. Every ordinary method of treatment was pursued, and the fever remained at 104°, and even reached 105°, with the various usual symptoms of delirium, etc. On auscultation and percussion, it was found that the lungs were both largely involved, and infiltration was nearly complete in one. Dispœna was excessively pronounced, weakness extreme; prognosis fatal. Everything having failed, it was decided, after consultation between Drs. Wiggins, of East St. Louis, Ill., and Lemen, of St. Louis, to try the serum, which I produce. The treatment began about the 9th day of June, 1895. The dose ranged between 20 to 40 ms. daily, and continued some six weeks, more or less regularly. The result was that the temperature decreased gradually and steadily after seven days treatment, to normal temperature, which was reached on the 22d of June, or thirteen days after the first injection. The patient rapidly and gradually picked up, gained flesh and strength, and is again at work. She is now healthier than ever before. She weighs 132 pounds, whereas she was emaciated to at least 80 pounds before treatment. The germs of tuberculosis have disappeared entirely, and every symptom of lung trouble is absent.

R. C. G. (St. Louis, Mo., aged 65 years); Dr. Lloyd Simpson, St. Louis, was physician in charge. Three consultants were called; diagnosis of all was acute tuberculosis and tuberculous pneumonia. Almost complete infiltration of the left lung, and extensive infiltration of the right. The patient, at the beginning of treatment, weighed between 150 and 160. Serum was continued three months, more or less regularly, every day, after which it was administered irregularly for about two months. This patient suffered, before the beginning of treatment, with imperfect kidney secretion, and had to be injected with care accordingly. The microscopical analysis of the sputum was made by Dr. Ravold, bacteriologist of the St. Louis Medical College, Dr. Geo. W. Cale, of the Woman's Medical College, before and at the beginning and during the course of treatment, and in every instance, until after some weeks of treatment, the bacilli of tuberculosis were found in quantities more or less profuse. The patient received injections of serum almost daily, in doses varying from 10 to 40 ms., for about three months, and irregularly thereafter for a month or more. At the end of three months, no bacilli were to be found in the sputum. The patient, who at the beginning of the treatment was in bed, picked up in flesh, and now weighs 224 pounds, and feels strong.

CHRONIC TUBERCULOSIS.

Mrs. H. R. (St. Louis, Mo.) consulted me in February, 1895. She had been ill two years, had had slight hemorrhages, was coughing very severely, expectorated muco-purulent material, occasionally tinged with blood. The sputum was profuse, and full of bacilli of tuberculosis. At the beginning of treatment, she evidenced infiltration in the apex of the right lung, between the third and fifth rib. There was softening and mucus rales about the middle of this area, and very pronounced interrupted breathing in both lungs. Weighed 90 pounds. On the regular dose of 30 ms. a day for four months, and then irregularly every third or fourth day, with a loss of three weeks one time, covering a period of six months, all told, Mrs. R. improved in flesh to the weight of 132 pounds, and became strong accordingly, developed a splendid appetite, and for the last three months her sputum, which is exceedingly scarce now and comes only when she is affected by cold, exhibits no bacilli of tuberculosis. She suffered a miscarriage and six weeks illness recently, but her lungs remain apparently sound. The physical lung symptoms which have existed at the beginning, have disappeared. The patient has recovered.

Mr. E. D. (St. Louis, Mo.), age 36; occupation, shipping clerk; history of glandular tuberculosis dating back eleven years. Had pneumonia four years previous to his examination at my office, May 16th, 1895. Had been declining six months; had had night sweats and fever, pain in left lung, back and front; pulse 108 at the time of examination; abnormal temperature ranged from 99 3-5 to 101; coughed chiefly in the morning; expectorated a slight yellowish matter; slept fairly on the right, but could not sleep on the left side; was too weak to attend to his duties constantly. There was a marked dullness in the left lower lobe and crepitus of the left apex over a lateral area of 4″, extending 3″ downward. There was interrupted breathing both sides. All these symptoms disappeared almost entirely in four months of treatment, consisting of 15 to 30 ms. of serum a day. Examination of the sputum made since, revealed no bacilli. Mr. D. is at work from twelve to fifteen hours per day, Sunday included, and feels strong, and is in good health. Weighs now 148 pounds, a gain of at least twenty-four pounds. He has recovered.

Mr. S. (St. Louis, Mo.) came to me to be examined in February, '95. He weighed about 145 pounds at the time. Had had very profuse hemorrhages at Hot Springs; had lost about sixty pounds from his regular weight, which was above normal for his size; was coughing a great deal night and day; expectorated a thick, yellowish matter, loaded with bacilli of tuberculosis and other microbes, and was rapidly declining and weakening. He was unable to perform any of his duties as a groceryman. His physical condition evidenced tuberculous affection of both lungs, particularly in the right. The lower half of the lung exhibited moist rales. The left was slightly involved in the same manner. These symptoms, after seven months of more or less regular treatment, which consisted of 20 ms. in the beginning and was increased to 30 and 40, and once in a while to 60 ms., almost entirely disappeared, with the exception of a slight interrupted breathing. Flesh was gained to the extent of 170 pounds, and strength seems now to be as good as ever. Mr. S. is now, and has been for the last six months, attending to his usual duties, working hard every day, and complains of nothing. He expresses the opinion that he is free from disease. He has recovered as much as any one with such lesions can expect to. He is as much cured as nature can effect a cure of such a case.

Mr. F. B. (St. Louis, Mo.); occupation, railroad clerk, working at night; had had bronchitis at the age of 14; had suffered from night emissions from early puberty. Previous health, cough, scarce; pain in the lower lobe of the left lung; temperature, 99 to 100. Physical examination evidenced dullness of the lower lobe, beginning at a line drawn below the nipple and extending down towards the base. Microscopical examination revealed the bacilli of tuberculosis. Mr. B. was treated from May 27th, 1895, to the middle of September, practically four months. All physical symptoms and evidences of tuberculosis have disappeared. He is now at work as before, and in good health. No bacilli have been found in the examination made since.

Mr. G. N. F. (St. Louis, Mo.); examined April 29th, 1895; age, 45; occupation, bookbinder. Has had a dry, hacking cough for two years; had had pneumonia at age of 18; congestion of lungs two years previous to his examination. February 7th, 1895, he had a hemorrhage which dragged him to bed, and at the beginning of the treatment he weighed 130 pounds; expectoration thick and yellowish; bacilli of tuberculosis numerous. Abnormal temperature, 99 3-5 to 101, infiltration of the left apex below second rib about 3″ downward and 4″ across, evidenced both anterially and posterially. Mr. F. was treated with tubercle anti-toxine from the beginning of May until the beginning of October almost every day, at the dose of 30 to 40 ms. Since then several examinations have been made, and no germs of consumption are to be found. On the other hand, physical symptoms have entirely disappeared. The patient weighs 146 pounds, has resumed work, and has entirely recovered.

Miss S. (Nashville, Tenn.) was admitted to the Sanitarium in May, 1895, and remained under treatment some three months. She came with a written diagnosis of pulmonary tuberculosis from her family physician, which was substantiated by microscopic and physical examination. The bacilli of tuberculosis were found in large numbers, and the patient was rapidly losing ground both in weight and strength, coughing considerably, particularly at night, and expectorating occasionally a yellowish matter, and sometimes a greenish matter. Night sweats had existed, and fever ranged from 99° to 102° F. She was treated with serum at doses ranging from 20 to 35 ms. After three months, she had gained ten pounds. She then migrated to Las Vegas, N. M., where she continued the treatment under Dr. Shaw, of the Santa Fe Railroad Hospital there, and her improvement continued. She, at first, lost flesh, but again increased, and every vestige of symptom seems to have disappeared, if I may judge from the reports sent me. The bacilli, and all the physical signs of lung disease, disappeared some months ago. She has recovered.

Miss G. A. (St. Louis, Mo.), age 19 years. Occupation, music and vocal student; had had influenza in Memphis six years before; dry, hacking cough for a year; weighed 123 pounds; hemorrhage four years previous to examination, September 26, 1895; larynx infiltrated; abnormal temperature, from 99½° to 101° F.; coughing considerable, and expectorated a yellowish matter. Bacilli of tuberculosis quite numerous. Heart disease evidenced by regurgitation. Treatment begun the last day of September, 1895. Injected very small doses, on account of her heart condition, that is, 10 to 20 ms. daily. At this time Miss A. weighed 132 pounds. The bacilli of tuberculosis are seldom present in her weekly examination, and when they appear they are very scarce, there being one or two in the field once in a while. Strength has been regained, and appetite is splendid. The patient is considered as having almost recovered, as there exists no longer the physical signs of infiltration and the first signs of breaking down, which existed at the beginning of treatment.

Mrs. N. (Chicago, Ill.), age 33, married, has a family of six or seven children; began treatment at the sanitarium May 19, 1895, and continued at the institution under the treatment for a period of two months, after which time she went away, and later reported as being free from disease. Her expectoration having ceased and cough being nil. The patient had been ill two years, had had pneumonia, following an operation for hemorrhoids. Having had no opportunity of making an examination since this report, I am unable to verify it with personal data.

Mr. V. (employee at our institution), age 31, a patient under the charge of Dr. L., had laryngeal and pulmonary tuberculosis. His condition had been declared, in writing, hopeless by a number of specialists in St. Louis, including all the leading ones. He has been treated under the special care of Dr. L. and myself for a period of about ten months, having received from 30 to 120 ms. a day. At the beginning of his treatment there existed infiltration and other lesions of the larnyx. He had lost his voice, weight and strength. He was in a hospital, unable to perform any work. He is now assisting in the care of some twenty-two horses in company with another man, working many hours every day in water and dust, and his appetite has improved and his strength keeps good. He is sensitive and susceptible to colds, but under the treatment with serum he has gained a condition which permits him to do all the labor that can be asked of almost any man.

Mrs. A. C., age 26, married, has had three children and two miscarriages, one recently. At the age of 14, she received a kick in the chest, at which point pain appeared frequently, and whenever the patient contracted cold. At her examination, infiltration was discovered over an area of about three inches in diameter on the right side below the breast, also a dullness in the left lung between the second and third ribs, extending about two inches downwards and two laterally. She had had the various symptoms of tuberculosis for some years, and dated the accidental incipiency of it fourteen years previous. She had had several hemorrhages. The active development of the disease dated three years previous to my examination, which occurred June 7, 1895. At that time she weighed 115 pounds; to-day she weighs 135 pounds. She had dispœna, expectorated a great deal, coughed almost unceasingly, had a very poor appetite. Now all these symptoms have disappeared, and her strength has increased so that she is able to perform her daily duties. The physical signs above mentioned have disappeared as completely as nature can heal such a case.

John H., age 48. Pulmonary tuberculosis two years. Prostrated in bed at city hospital. Cavity, contracted lung, bacilli numerous. Mother died of phthisis; cough aggravating. Injections 30 ms. daily; begun December 1, 1894; continued for three months regularly. Weight increased 6½ pounds first two months. Has since been under treatment irregularly. Has been at work over a year, grooming from twelve to twenty horses, ploughing and farming generally part of the time. Bacilli absent at present. Recovery.

Miss Y., age 18. Pulmonary tuberculosis, first stage. Lost ten pounds. Infiltration in and dullness over area of about three inches in diameter between second and sixth ribs, in right lung. Cough persistent at night and expectoration profuse in the morning; bacilli present.

Miss Y. was treated over four months almost daily, at a dose of 30 ms., hypodermically. To-day she has more than gained her normal weight, is absolutely free from cough, and the last symptom of pulmonary tuberculosis has disappeared. Bacilli absent.

SURGICAL TUBERCULOSIS.

B. McG., age 18 years 3 months, had been suffering with joint and bone tuberculosis for seven years, and had ten operations performed on different parts of his body to open abscesses and to open necrosed bone. The seat of his trouble was the right hip-joint, but was giving him trouble on every limb. The left tibia was much involved, having at one time eight openings discharging a tubercular pus. The hip had three openings that would heal and open alternately, and one that was open contintinually for seven years. He had an abscess on each arm, one of the sterum, one of the index finger of the right hand, a tubercular nodule in the skin of the scrotum, an abscess near the apex of the left scapula and two on the lower jaw. After the continued efforts of Dr. J., of Waverly, Ky., who had for consultants two physicians of the same State, to cure the patient had failed, it was decided to let nature take its course. He was without aid for two years. In the fall of 1894 he was put in care of Dr. B., of St. Louis, who performed an operation to remove necrossed bone from the thigh and tibia, thinking these openings would heal. This having failed, he decided to try the serum treatment, and was taken in charge by Dr. G. W. C., of St. Louis, Mo. He began the treatment in March, 1895, at which time he had four abscesses discharging a characteristic tubercular pus, and two places that afterwards opened. He had daily injections of serum in doses of 20 to 30 ms., and at the close of six month's treatment five of these abscesses had closed, he had gained 10 pounds, and was without temperature. He is working steadily, and is still gaining weight, and hopes for a cure. The last and only opening is on the thigh, and dead bones have been located, which is the cause of its remaining open. Up to the present date he has gained 16 pounds, has a good appetite, and is enjoying good general health.

